# Hybrid Nanoparticles of Poly (Methyl Methacrylate) and Antimicrobial Quaternary Ammonium Surfactants

**DOI:** 10.3390/pharmaceutics12040340

**Published:** 2020-04-10

**Authors:** Beatriz Ideriha Mathiazzi, Ana Maria Carmona-Ribeiro

**Affiliations:** Biocolloids Laboratory, Departamento de Bioquímica, Instituto de Química, Universidade de São Paulo, Av. Prof. Lineu Prestes 748, 05508-000 São Paulo, Brazil; bemathi@usp.br

**Keywords:** hybrid nanoparticles, biocompatible polymer, antimicrobial amphiphiles, dynamic light scattering, scanning electron microscopy, cell viability from counting of colony-forming unities, antimicrobial activity of nanoparticles, *Escherichia coli*, *Staphylococcus aureus*, *Candida albicans*

## Abstract

Quaternary ammonium surfactants (QACs) are microbicides, whereas poly (acrylates) are biocompatible polymers. Here, the physical and antimicrobial properties of two QACs, cetyl trimethyl ammonium bromide (CTAB) or dioctadecyl dimethyl ammonium bromide (DODAB) in poly (methyl methacrylate) (PMMA) nanoparticles (NPs) are compared to those of QACs alone. Methyl methacrylate (MMA) polymerization using DODAB or CTAB as emulsifiers and initiator azobisisobutyronitrile (AIBN) yielded cationic, nanometric, homodisperse, and stable NPs. NPs’ physical and antimicrobial properties were assessed from dynamic light scattering (DLS), scanning electron microscopy, and viability curves of *Escherichia coli*, *Staphylococcus aureus*, or *Candida albicans* determined as log(colony-forming unities counting) over a range of [QACs]. NPs were spherical and homodisperse but activity for free QACs was higher than those for QACs in NPs. Inhibition halos against bacteria and yeast were observed only for free or incorporated CTAB in NPs because PMMA/CTAB NPs controlled the CTAB release. DODAB displayed fungicidal activity against *C. albicans* since DODAB bilayer disks could penetrate the outer glycoproteins fungus layer. The physical properties and stability of the cationic NPs highlighted their potential to combine with other bioactive molecules for further applications in drug and vaccine delivery.

## 1. Introduction

Biocompatible synthetic or natural polymers can improve body functions without interfering with its normal functioning or triggering side effects [1,2,3,4,5,6,7]. They have been useful in tissue culture, tissue scaffolds, implants, artificial grafts, wound dressings, controlled drug release, prosthetic replacements for bones, dentistry, etc [8]. Some examples of biocompatible polymers are poly (lactic-co-glycolic acid) [9], poly (ε-caprolactone) (PCL) [10], poly (lactic acid) [11], poly (3- hydroxybutyrate-co-3-hydroxyvalerate) (PHBV) [12], chitosan [13,14], cellulose [15], and poly (acrylates) including poly (methyl methacrylate) (PMMA) [6,16,17,18]. Among the poly (acrylates), several cationic latexes and coatings obtained from additives such as cationic surfactants, lipids, antimicrobial polymers or co-polymers have been described [19,20,21,22,23,24,25]. In the case of PMMA, the discovery of PMMA biocompatibility occurred when an optical technician placed a PMMA scleral lens on his eye and found that it could be well tolerated. The first PMMA patent appearing in 1948 [16] was acquired by Bausch & Lomb in 1972 [26].

In our laboratory, the good compatibility between PMMA and some antimicrobial quaternary ammonium compounds (QACs) was first described both for PMMA/QAC coatings [19] and PMMA/QACs nanoparticles (NPs) [27]. The QACs were apparently able to impart their antimicrobial activity [28,29,30,31] to the PMMA/QACs assemblies [19,27]. In the absence of PMMA, QACs in water dispersion self-assemble to yield micelles or bilayers depending on the QAC chemical structure and molecular shape [32,33]. Dioctadecyl dimethyl ammonium bromide (DODAB) assembles in water solution as bilayer vesicles or bilayer fragments (BF) depending on the dispersion method; sonication disrupts DODAB vesicles producing BF [34,35,36]. Cetyltrimethylammonium bromide (CTAB) assembles in water solution as micelles above the critical micellar concentration [32]. Ion–dipole interactions between the quaternary ammonium in DODAB and the carbonyl moiety in PMMA plus other weak but cooperative van der Waals interactions led to hybrid materials where DODAB was found well dispersed in the polymeric matrix [19]. This allowed for combining the bactericidal DODAB property [29,30,31,37,38,39,40,41,42] with excellent DODAB immobilization and distribution in the PMMA polymeric network [19]. Later on, determining the effect of QAC chemical structure on the antimicrobial activity of PMMA/QAC coatings revealed that DODAB and CTAB behaved differently; CTAB diffused through the film and reached bacteria in the outer medium, whereas DODAB impregnation in the polymeric matrix killed bacteria upon contact without leakage [20].

**Scheme 1:**
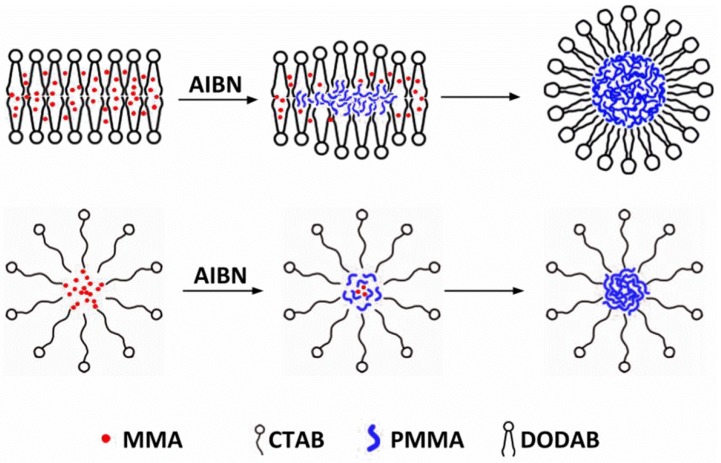
Emulsion polymerization of methyl methacrylate (MMA) initiated by azobisisobutyronitrile (AIBN) using dioctadecyl dimethyl ammonium bromide (DODAB) or cetyltrimethylammonium bromide (CTAB) as emulsifiers. The cross sections of DODAB bilayer fragments (BF) and CTAB micelles were schematically represented as loaded with the MMA monomer (in red) before adding AIBN to initiate the polymerization.

In this work, PMMA/QAC NPs synthesized by emulsion polymerization of methyl methacrylate (MMA) using azo-bis-isobutyronitrile (AIBN) as initiator and DODAB BF or CTAB as emulsifiers were evaluated regarding their physical properties and antimicrobial activity as compared to the free QACs. Scheme 1 illustrates the emulsion polymerization of MMA in the presence of DODAB BF or CTAB using AIBN as the initiator.

Over a range of MMA or QAC concentrations, 0.4 M MMA and 2 mM QAC yielded optimal physical properties for the PMMA/DODAB and PMMA/CTAB NPs (nanometric size, low polydispersity, high and positive zeta-potential, high yield, and high colloidal stability). Inhibition halos by CTAB and PMMA/CTAB NPs against bacteria and yeast showed that PMMA/CTAB NPs behaved as reservoirs for the release of CTAB with time after dialysis. CTAB was able to move both through the dialysis membrane and the agar to inhibit microbial growth. In contrast, DODAB incorporated in the PMMA polymeric matrix did not move in the agar and did not cross the dialysis membrane. For PMMA/DODAB NPs or DODAB BF, inhibition halos were not observed due to the lack of DODAB diffusion in the agar medium. The incorporation of QACs in the PMMA/QAC NPs reduced antimicrobial activity in comparison to the QACs in dispersion. CTAB was the most active microbicidal agent against the three microbes tested (*E. coli*, *S. aureus*, and *C. albicans*) reducing cell viability countings by 7 logs at submicellar concentrations. CTAB mobility in hydrated medium favored its electrostatic interaction and penetration through the microbes’ cell wall and membrane, imparting a lytic effect on the cells. DODAB BF revealed an important fungicidal activity against *C. albicans* not described before; similar to rod-like copolymers assemblies, the disk-like cationic DODAB BF possibly entered the outer glycoproteins layer of the fungus penetrating the cell and causing a 5-log reduction in yeast viability. Besides applications in antimicrobial chemotherapy, PMMA/QAC NPs here described may find interesting uses also as immunoadjuvants due to their nanometric size, positive zeta-potential, narrow size distribution, and high colloidal stability. They are expected to combine well with oppositely charged antigens, such as proteins [43], peptides [44,45], or DNA [46,47,48] for subunit vaccines design as many cationic adjuvants do.

## 2. Materials and Methods

### 2.1. Materials

Methyl methacrylate (MMA), hexadecyltrimethyl ammonium bromide (CTAB) dioctadecyldimethyl ammonium bromide (DODAB), azobisisobutyronitrile (AIBN), agarose, Mueller-Hinton agar (MHA), D-glucose, NaCl, ethanol 99.9%, and cellulose acetate membranes with a molecular weight cutoff around 12,400 g/mol were obtained from Sigma-Aldrich (Darmstadt, Germany) and used without further purification.

### 2.2. Preparation of CTAB or DODAB Dispersions in Water Solution

CTAB and DODAB are quaternary ammonium amphiphiles (QACs) that were separately weighted and added to a 1-mM NaCl solution (pH 6.3) to yield a stock dispersion at 10 mM amphiphile. Whereas CTAB yielded a homogeneous and transparent dispersion, typical of its assembly in water as micelles, DODAB had to be dispersed ultrasonically with a macroprobe. The DODAB BF were obtained from the DODAB powder added to the aqueous 1 mM NaCl solution dispersed by sonication with tip at 85 W for 15 min above 47 °C, before centrifuging the dispersion for precipitation of titanium ejected from the tip (9300× *g* for 1 h at 4 °C). This yielded the DODAB BF as a somewhat turbid dispersion. These DODAB dispersions were previously characterized as containing open bilayer fragments that did not enclose a water compartment, were nano-sized, and able to incorporate hydrophobic drugs in the bilayer or antimicrobial peptides or polymers [34,35,44,45,49,50,51,52,53,54]. Both CTAB and DODAB dispersions were diluted from the stock dispersions to obtain the final desired concentrations. The analytical CTAB or DODAB concentrations were determined from halide microtitration [55,56].

### 2.3. Synthesis of Waterborne PMMA/QACs Nanoparticles (NPs) by Emulsion Polymerization

The PMMA/CTAB or PMMA/DODAB hybrid and polymeric NPs were obtained by emulsion polymerization of MMA using AIBN as the initiator of the MMA polymerization, similar to the synthesis previously described using potassium persulfate (KPS) as the initiator [27]. The synthesis protocol was similar to the one previously described for the synthesis of PMMA/PDDA NPs using AIBN as the initiator [21,22,23]. In order to eliminate oxygen, a flow of nitrogen gas was applied for a few minutes to 10 mL DODAB or CTAB dispersions in 1 mM NaCl added of the desired MMA concentration and 0.0036 g AIBN. The reaction mixture inside the glass tube was then closed with a cap, heated, and kept at 80 °C in a water bath for 1 h under periodic vortexing. Thereafter, the capped tube containing the reaction mixture was withdrawn from the water bath and allowed to reach room temperature. The dispersions thus obtained were purified by dialysis against 2 L of ultrapure water (3×) for 24 h. Particle characterization took place after dialysis using dilutions of the original dispersion in 1 mM aqueous NaCl solution.

### 2.4. Determination of Sizes, Zeta-Potentials, and Polydispersity of PMMA/QAC Dispersions by Dynamic Light Scattering (DLS)

Size distributions, zeta-average diameters (Dz), and zeta potentials (ζ potentials) were obtained by dynamic light scattering (DLS) using a Zeta plus−Zeta potential Analyzer (Brookhaven Instruments Corporation, Holtsville, NY, USA) equipped with a 677-nm laser with measurements at 90°. The polydispersity of the dispersions was determined by dynamic light scattering (DLS) following well-defined mathematic equations [57]. The mean hydrodynamic diameters (mean Dz) were obtained from the log-normal distribution of the light-scattering intensity curve against Dz. The ζ potentials were determined from the electrophoretic mobility (μ) and the Smoluckowski equation, ζ = μη/ε, where η and ε are the viscosity and the dielectric constant of the medium, respectively. Samples that underwent the DLS measurements were usually diluted from the original dispersions for optimal readings (50–100 μL PMMA/QAC dispersions in 2 mL of 1 mM NaCl). All measurements were performed in the DLS apparatus took place at 25 ± 1 °C.

### 2.5. Visualization and Morphology of PMMA/QAC NPs from Scanning Electron Microscopy (SEM)

Scanning electron microscopy (SEM) was performed using Jeol JSM-7401F equipment. Silicon <100> wafers were from Silicon Quest (Santa Clara, CA, USA) with a native oxide layer approximately 2 nm thick and used as substrates for casting the PMMA/QAC dispersions. The Si wafers with a native SiO_2_ layer were cut into small pieces of ca 1 cm^2^, cleaned with ethanol, and dried under an N_2_ stream. Samples of 0.050 mL PMMA/QAC dispersions were deposited on clean Si/SiO_2_ wafers and dried overnight in a desiccator. Then, the coatings were covered with a thin layer of gold before SEM analyses, as required for contrast and visualization. Mean diameters (D) for dry NPs were evaluated from ImageJ software Version 1.52u for 100 particles and presented as a mean value.

### 2.6. Microorganisms Cultures and Effect of CTAB, DODAB, or PMMA/QAC NPs on Cell Viability in the Presence of the Cationic Amphiphiles Solutions or Dispersions

*Escherichia coli* ATCC (American Type Culture Collection) 25922, *Staphylococcus aureus* ATCC 29213 or *Candida albicans* ATCC 90028 were cultured from previously frozen stocks (kept at −20 °C in the appropriate storage medium). Each microorganism was reactivated separately, seeded by streaking technique on the plates of Mueller-Hinton agar, and incubated for 18–24 h at 37 °C. The turbidity of either bacteria or fungus suspensions was adjusted according to tube 0.5 of the McFarland scale at 625 nm in isotonic 0.264 M D-glucose solution. The 0.264 M D-glucose solution was used instead of any culture medium because cationic molecules are inactivated by the relatively high ionic strength or negatively charged molecules, such as amino acids and polysaccharides. For the determination of cell viability, 0.1 mL of the cell suspensions (around 10^7^–10^8^ colony-forming unities per mL, CFU.mL^−1^) were mixed with 0.9 mL of NPs dispersions diluted in the same D-glucose solution and interacted for 1 h. Thereafter, aliquots of 0.1 mL were withdrawn and either directly plated or diluted 10 to 10^6^ times before plating on MHA plates. The plates were incubated at 37 °C for 24 h. The CFU were counted and plotted in a logarithmic or percentage scale as a function of QAC concentration (mM). When no counting was obtained, since the log function does not exist for zero, the CFU counting was taken as 1 so that log CFU.mL^−1^ could be taken as zero.

### 2.7. Determination of Growth Inhibition Zones by PMMA/QAC NPs

*Escherichia coli* ATCC (American Type Culture Collection) 25922, *Staphylococcus aureus* ATCC 29213, and *Candida albicans* ATCC 90028 from previously frozen stocks kept at −20 °C in an appropriate storage medium were grown as described above, and the bacterial suspension prepared in 0.264 M D-glucose had its turbidity adjusted according to 0.5 of the McFarland scale, as previously described. A softer growth medium containing 2.3% Muller-Hinton broth and 0.64% agar was prepared and sterilized in steam autoclave at 121 °C for 20 min. In 50 mL of this growth medium, 0.5 mL of the microorganism suspension was added and then carefully homogenized. Plates containing MHA were previously prepared and used to place micropipette tips with their bases positioned on the MHA to form wells with a 9 mm diameter. Before withdrawing the tips, ca. 20 mL of the microorganism culture in the soft MHA was allowed to harden. After agar hardening, the tips were removed and, in each well, 100 μL of the QAC dispersions at 0.01, 0.1, 0.2, 0.5, 1, 1.5, 2, and 2.5 mM or of the NPs dispersions were added for incubation 18–24 h at 37 °C for determining the inhibition zones surrounding the wells. From the comparison between inhibition zones for the standard QAC dispersions and the PMMA/QAC NPs, the QAC concentration in the NPs dispersions was estimated.

### 2.8. Determination of QAC Concentration from Halide Microtitration

Bromide is the counterion of DODAB and CTAB. Similar to chloride, bromide can be determined by halide microtitration [55], as given in a detailed protocol for halide microtitration [56].

## 3. Results and Discussion

### 3.1. Synthesis of PMMA/QACs NPs by Emulsion Polymerization and their Physical Characterization from SEM and DLS

**Figure 1:**
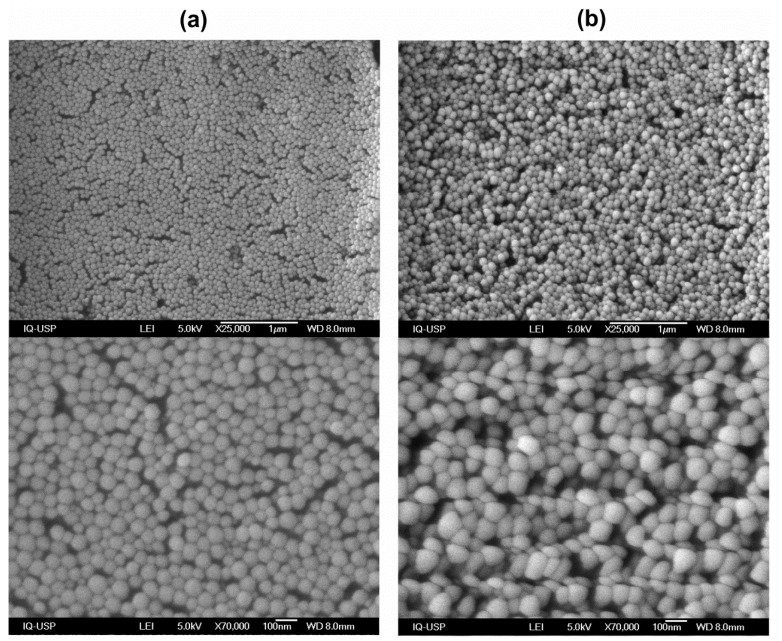
Scanning electron micrographs (SEM) of original dispersions of poly methyl methacrylate /dioctadecyldimethylammonium (PMMA/DODAB) (**a**) or PMMA/CTAB nanoparticles (NPs) on silicon wafers (**b**). The magnification was either 25,000× (on the top) or 70,000× (on the bottom).

PMMA/QAC NPs’ morphology, size, and homogeneity were assessed by SEM. Two different PMMA/QAC NPs were synthesized and observed after drying under SEM (Figure 1). The QAC was DODAB (Figure 1a) or CTAB (Figure 1b). Both syntheses employed 0.4 M MMA, 2 mM QAC, 0.0036 g AIBN and 1 mM NaCl in 10 mL of reaction mixture. The NPs were spherical, displayed a narrow size distribution and were homo dispersed. PMMA/DODAB and PMMA/CTAB mean diameters after drying (D) were obtained for at least 100 particles from the ImageJ software as 56 ± 7 and 85 ± 11 nm, respectively (Figure 1).

The mean diameter of dry NPs (D) was compared to the one of NPs in water dispersion given by dynamic light scattering (DLS) as the mean hydrodynamic diameter (Dz) (Table 1). 

For NPs obtained from 0.4 M MMA, 2 mM DODAB, or CTAB using 0.0036 g AIBN, the mean hydrodynamic diameters (Dz) were 75 ± 1 and 81 ± 1 nm for PMMA/DODAB and PMMA/CTAB NPs, respectively. One would expect that the Dz values from DLS would be higher than D from SEM due to an eventual hydration layer surrounding each NP. Indeed, this occurred for the PMMA/DODAB NPs but did not occur for the PMMA/CTAB NPs. It is possible that the PMMA/CTAB NPs exhibited a less hydrophilic surface at the particle/water interface than the PMMA/DODAB NPs. This was possibly due to the mobile character of the CTAB molecules leaving the nanoparticles to the bulk water and the large affinity of the DODAB molecules for the PMMA polymeric matrix [19,20]. Whereas DODAB would improve the NP affinity for the surrounding water and the hydration layer, this would not be very significant for CTAB so that, for CTAB, the dry, dehydrated diameter D would not be significantly lower than the hydrodynamic diameter Dz. D was 56 ± 7 nm for PMMA/DODAB NPs and 85 ± 11 nm for PMMA/CTAB NPs.

Curiously, DODAB behaved as a better emulsifier than CTAB during the emulsion polymerization process remaining in the PMMA/DODAB NPs after polymerization due to its higher affinity for PMMA than the one exhibited by CTAB [19,20]. At this point, one should recall the nanostructures present in DODAB and CTAB dispersions in water: the DODAB bilayer fragments (DODAB BF) with Dz inside the 56–67 nm range, as determined by DLS or SEM [44,58,59] or CTAB micelles with 10–20 nm of diameter determined by small-angle neutron scattering (SANS) [60]. IT is possible that the higher amount of DODAB in DODAB BF as compared to the amount of CTAB in the micelle improved the emulsifying effect of DODAB and resulted in a smaller NP size for the PMMA/DODAB NPs than the one for the PMMA/CTAB NPs. Since CTAB has only one hydrocarbon chain, its hydrophilic–hydrophobic balance is larger than the one for DODAB with two long hydrocarbon chains. The resulting emulsifying effect during NP synthesis promoted by CTAB was inferior to the one promoted by the DODAB BF so that sizes for the resultant NPs were larger than those synthesized with DODAB BF.

### 3.2. Effects of MMA Concentration, QAC Concentration, and Initiator Type on Physico-Chemical Properties of PMMA/QAC NPs Obtained by Emulsion Polymerization

Over a range of [MMA] varying from 0.1 to 1 M MMA, PMMA/QAC dispersions exhibited variable colloidal stability after synthesis and before dialysis. Above 0.4 M MMA, at 2 mM QAC, aggregation and precipitation took place so that only the supernatants were dialyzed and used for DLS and solid contents analysis. From 0.1–0.4 M MMA, no precipitation took place after synthesis and before dialysis.

**Figure 2:**
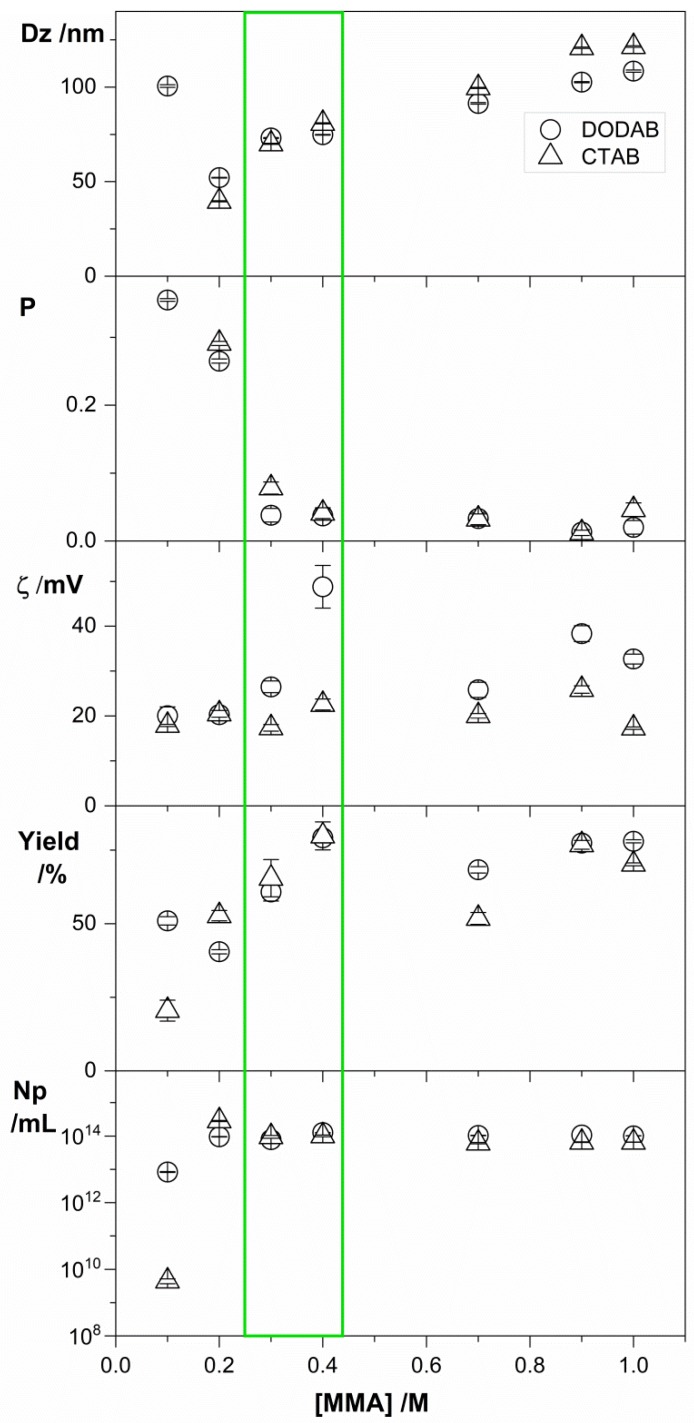
Physical properties of PMMA/QAC NPs obtained by emulsion polymerization over a range of [MMA] at 2 mM DODAB (○) or CTAB (∆). All dispersions were exhaustively submitted to dialysis in pure water before measurements. Above 0.4 M MMA, poor colloidal stability was depicted from the visual observation of precipitated material; the measurements were performed with the dialyzed supernatants. Below 0.3 M MMA, low MMA concentrations resulted in low conversion (yield %), high polydispersities (P), and comparatively low zeta-potentials (ζ). The green rectangle indicates the region of [MMA] yielding stable PMMA/QACs NPs at high conversion rates, maximal particle number densities (N_p_), low P (0.03–0.06), high ζ (20–50 mV), and low Dz (70–75 nm).

Table 2 and Figure 2 show the effect of [MMA] on the physical properties of PMMA/QAC NPs obtained by emulsion polymerization at 2 mM QAC.

The data on Table 2 reveal that increasing [MMA] from 0.1 to 0.4 M at 2 mM DODAB, slightly increased the Dz for the NPs, increased ζ, reduced P, increased the solid contents and conversion of monomer into polymer, and slightly increased number density (N_p_) for the NPs, in absence of aggregation. Above 0.4M MMA, poor colloidal stability associated with low zeta-potential was depicted as precipitation at the bottom of the assay tubes (Table 2). For PMMA/CTAB NPs, similar behavior took place for the physical properties except for the zeta-potential that remained low and approximately constant 17–23 mV. These results for the compared zeta-potentials for PMMA/DODAB and PMMA/CTAB NPs, again, suggest that increasing [MMA] leads to increased incorporation of DODAB in the NPs, whereas the incorporation of CTAB remains low and practically unaffected by the increased amount of solid contents. 

Figure 2 gives an overview of the effect of [MMA] on the physical properties of the PMMA/DODAB and PMMA/CTAB NPs’ dispersions. The green rectangle emphasizes the NPs obtained with desirable characteristics, such as low size, low polydispersity, high zeta-potential, high yield, high particle number density (Np), and high colloidal stability (absence of aggregates and precipitates). The synthesis performed at 0.4 M MMA was selected as the one yielding the optimal NPs to be analyzed regarding their antimicrobial properties. One should notice that at 0.1 M [MMA] in the presence of DODAB BF, the high P and large sizes obtained might be explained from the intrinsically high polydispersity of the DODAB BF; polymerization took place inside the bilayer fragments, and NPs acquired their polydispersity.

The effect of [QAC] on the physical properties of the PMMA/QAC NPs is shown in Table 3. Increasing [QAC] reduced Dz, decreased the zeta-potential of PMMA/DODAB NPs and increased the one of PMMA/CTAB NPs, increased the polydispersity, and barely affected conversion or particle number density. The highest zeta-potentials for PMMA/DODAB NPs occurred at the lowest [DODAB], meaning that DODAB imparted high positive charges to the NPs but preferred to interact with other DODAB BF in dispersion when [DODAB] increased. For PMMA/CTAB, similar behavior to the one of PMMA/DODAB NPs was found except for the zeta-potential that decreased with [DODAB] and increased with [CTAB]. This means that the low affinity of CTAB for PMMA again explained the low incorporation of CTAB to this polymer over a range of low CTAB concentrations. Only when [CTAB] increased did it become possible to cause an increase in the zeta-potential of the PMMA/CTAB NPs from 15 to 40 mV due to increased CTAB incorporation in the NPs (Table 3). Increasing [QAC], affected the polydispersity that also increased for both types of NPs. QACs indeed tend to increase the size of their aggregates with increase in their concentration or on the ionic strength of the medium [60,61,62]. The selected concentration of MMA was 0.4 M because, above 0.4 M MMA, poor colloidal stability was depicted from a visual observation of precipitated material; in this case, the measurements were performed with the dialyzed supernatants. Below 0.3 M MMA, low MMA concentrations resulted in low conversion (yield %), high polydispersities (P), and comparatively low zeta-potentials (ζ). The green rectangle indicates the region of [MMA] yielding stable PMMA/QACs NPs at high conversion rates, maximal particles number densities (N_p_), low P (0.03–0.06), high ζ (20–50 mV), and low Dz (70–75 nm).

**Figure 3:**
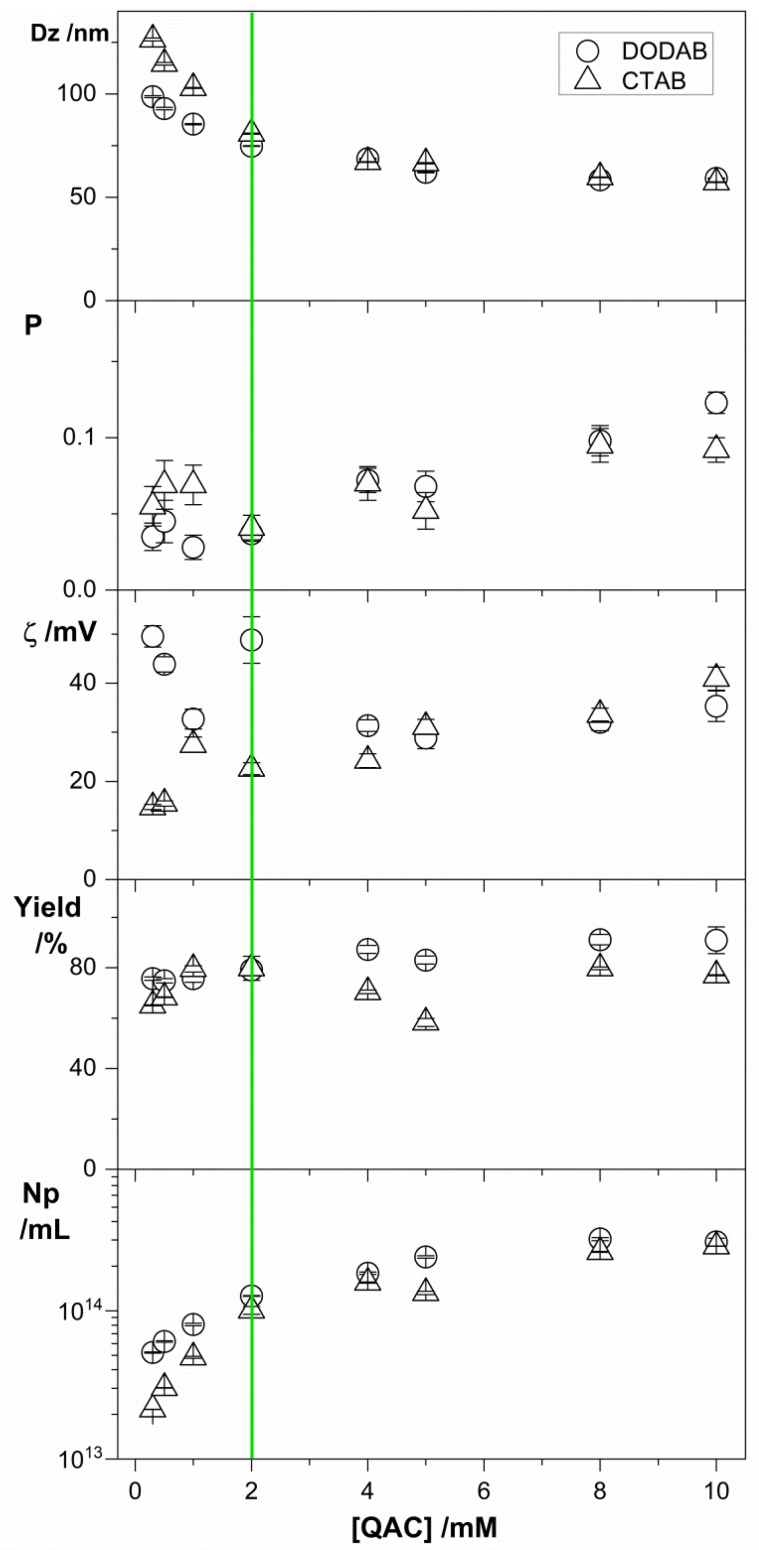
Physical properties of PMMA/QAC NPs obtained by emulsion polymerization over a range of [QAC] at 0.4 M MMA during particle synthesis where QAC is DODAB (○) or CTAB (∆). All dispersions were exhaustively submitted to dialysis in pure water before measurements.

Figure 3 showed the desired properties for the PMMA/QAC dispersions at 2 mM QAC (green line), which was the concentration selected for the evaluation of antimicrobial activity. At 2 mM DODAB or CTAB, there was low size, low polydispersity, high zeta-potential, high yield, high Np and good colloidal stability for the PMMA/QAC NPs.

Two initiators were compared for the NPs synthesis: potassium persulfate (KPS) and AIBN. The initiator effect on NP properties is shown in Table 4. The MMA concentration selected for the comparison was identical to the one previously used in [27], where NPs had been synthesized with 0.56 M MMA using KPS as initiator.

Imparting the positive charge on the NPs was a difficult task in the presence of the negative sulfate charges on the PMMA/ DODAB particles coming from the synthesis with KPS as the initiator; at 2 mM DODAB during the NPs synthesis, the zeta-potential was still negative (ζ = −10 ± 1 mV) and the particles were large (Dz = 1260 ± 43) and very polydisperse (P = 0.370), suggesting poor colloidal stability (Table 4). Naves and coworkers used large concentrations of the QACs, such as ca. 10 mM DODAB, to revert the KPS effect and obtain positively charged NPs [27]. AIBN was a much more convenient initiator since, when using only 2 mM DODAB, the dispersion contained small NPs (Dz = 89 ± 1) that were positively charged (ζ = +45 ± 2 mV) and homodispersed (P = 0.027 ± 0.010). Similar results were obtained from the comparison between PMMA/CTAB NPs synthesized with KPS and PMMA/CTAB NPs synthesized with AIBN (Table 4). In summary, at 2 mM QAC, the comparison between NPs synthesized using KPS or AIBN indicated that the low sizes, the high positive zeta-potentials, and the low polydispersities occurred only using AIBN.

### 3.3. Incorporation of QACs in the PMMA/QAC NPs

DODAB and CTAB have a different hydrophobic–hydrophilic balance, as depicted from their chemical structure. The double-chained DODAB tends to prefer more hydrophobic environments than the single-chained CTAB. The determination of QAC incorporation in the PMMA polymeric matrix of the NPs showed the higher incorporation of DODAB in comparison to the one of CTAB (Table 5).

A CTAB control solution with 2.5 mM CTAB before dialysis permeated the dialysis membrane almost completely, yielding 0.1 mM CTAB just after dialysis (Table 5). DODAB BF at 2 mM DODAB, on the contrary, did not permeate the dialysis bag. Similarl to CTAB, NaCl permeated the dialysis membrane almost completely. On the third day after dialysis, the supernatants of centrifuged PMMA/DODAB dispersions contained 1.3 mM DODAB, suggesting that 0.7 mM DODAB was still incorporated by the PMMA/DODAB NPs. PMMA/CTAB NPs dispersions had contents of QAC determined after dialysis and centrifugation, revealing the absence of CTAB in the supernatants just after dialysis but its presence in the supernatant 3 days after dialysis showing its low affinity for the PMMA/CTAB NPs. In contrast, 3 days after dialysis, 0.5 mM CTAB was determined in the supernatant of PMMA/CTAB NPs showing CTAB leakage from the PMMA/CTAB NPs just after dialysis (Table 5). In summary, DODAB incorporation in the NPs was substantial, whereas the one of CTAB was transient and possibly almost lost after dialysis.

**Figure 4:**
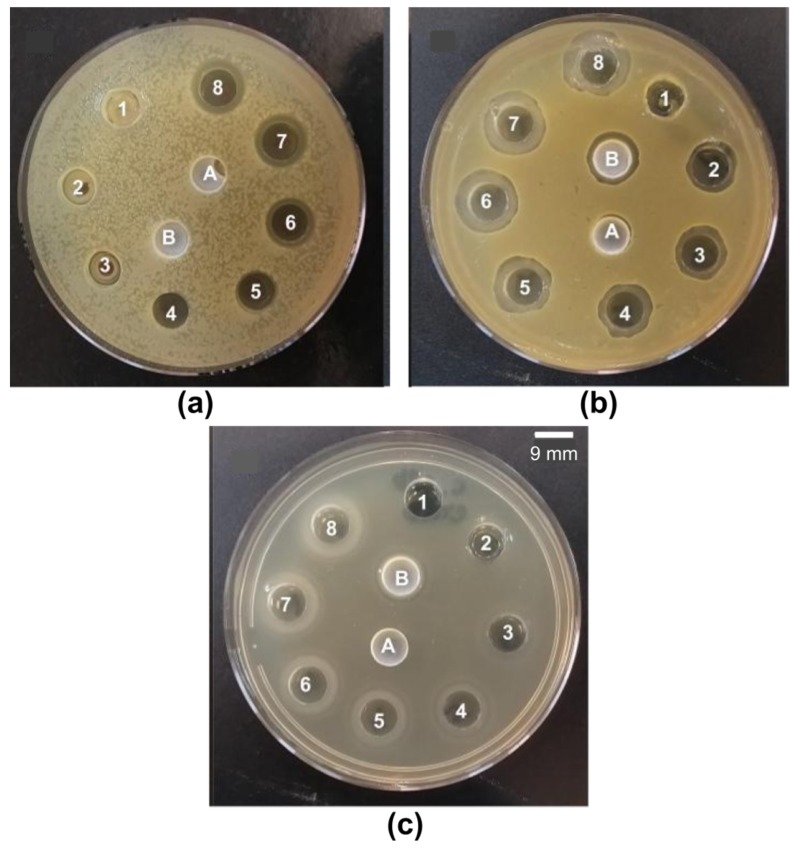
(**a**) *E. coli*, (**b**) *S. aureus*, and (**c**) *C. albicans* inhibition halos induced by CTAB alone (numbered from 1 to 8) or by PMMA/CTAB dispersions after (A) or before dialysis (B). From 1 to 8, [CTAB] inside the wells was 0.01, 0.2, 0.3, 0.5, 1.0, 1.5, 2.0, and 2.5 mM.

The evaluation of inhibition halos by CTAB against bacteria and yeast is in Figure 4. Just after dialysis, CTAB was not found in the supernatants of PMMA/CTAB NPs. This contrasted with 0.5 mM CTAB found in the supernatant 3 days after dialysis (Table 5). PMMA/CTAB NPs behaved as a reservoir for the release of CTAB with time after dialysis. The experiment using CTAB to determine the inhibition halos also showed that CTAB is able to move through the agar to inhibit microbial growth. Due to this property, inhibition halos against seeded bacteria and fungus could be determined on Petri dishes as a function of [CTAB] over a range of [CTAB] (0.01–2.5 mM CTAB) (Figure 4). Inhibition halos occurred for PMMA/CTAB NPs before (B) dialysis but did not occur just after dialysis (A), confirming the momentaneous absence of CTAB in the NPs supernatant. Estimated [CTAB] in the NPs’ supernatant before dialysis resulted from the similarity with halo 3 against *S. aureus* (Figure 4b) or halo 3 against *C. albicans* (Figure 4c), yielding 0.3 mM CTAB outside the NPs. If the added CTAB during particle synthesis was 2 mM, after synthesis, 1.7 mM was incorporated in the NPs and 0.3 mM was in the outer solution. However, the NPs dialysis after synthesis eliminated non-incorporated CTAB from the dispersions, as depicted from the absence of halo in A (Figure 4). Regarding the antimicrobial activity that will be determined just after dialysis, one will have to consider 1.7 mM as the CTAB concentration in the NPs.

For PMMA/DODAB NPs, the experiment based on inhibition halos was not possible since DODAB BF are not able to diffuse in the agar medium as reported before [23].

### 3.4. Antibacterial and Antifungal Activity of QACs and PMMA/QAC NPs

A proper evaluation of the antimicrobial activity involves determining CFU counting over a range of [QAC] and expressing the CFU countings on a logarithmic scale so that the effective potency of the QACs becomes evaluated over ample range of magnitude.

**Figure 5:**
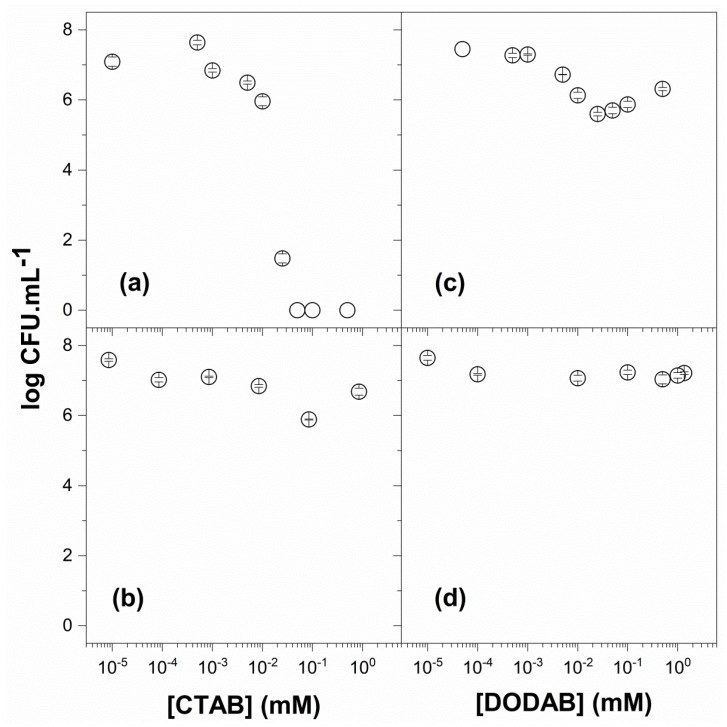
Viability of *Escherichia coli* in the presence of CTAB (**a**), PMMA/CTAB (**b**), DODAB (**c**), and PMMA/DODAB dispersions (**d**). The NP dispersions of PMMA/CTAB and PMMA/DODAB were used after dialysis. The counting of colony-forming unities (CFU) was expressed on a logarithmic scale.

Figure 5 shows the cell viability of *Escherichia coli* in the presence of CTAB (a), PMMA/CTAB NPs (b), DODAB BF (c), and PMMA/DODAB NPs (d). Just after dialysis, the inhibition halo experiment yielded an absence of free CTAB in the supernatant of PMMA/CTAB NPs and 1.7 mM CTAB in the NPs. This [CTAB] initially incorporated in the NPs decreased *E. coli* viability by 1.5 logs (Figure 5b), in contrast to free CTAB that reduced viability by 7 logs (Figure 5a). CTAB exhibited a remarkable activity against *E. coli* that could not be identified before from experiments expressing only % cell viability (limited to only 2 logs at most). CTAB incorporation in the PMMA/CTAB NPs reduced substantially its activity but may be useful for controlled release in biomedical prosthetic devices [63] or agar-based hydrogels [64].

The CTAB mechanism of action involves reaching the bacterial cell membrane causing its disruption and cell lysis [65]. Therefore, the large reduction in CTAB activity was due to its location in the PMMA/CTAB NPs instead of moving freely in the bulk solution to interact with the bacteria. In another report, CTAB adsorbed by BiOBr nanosheets showed lower toxicity than the same level of free CTAB, which was attributed to the adsorption or hindering effect of BiOBr nanosheets [66]. PMMA/CTAB NPs in this work released 0.5 mM CTAB on the third day after dialysis from an initial concentration in the NPs of 1.7 mM CTAB (Table 5; Figure 4). This behavior was similar to the one of the inorganic BiOBr nanosheets incorporating CTAB that exhibited a slow and sustained release of CTAB or benzalkonium chloride for 8 h [66].

Among the biocompatible polymers, PMMA was used in combinations with CTAB or DODAB to prepare spin-coated films able to kill bacteria upon release to the medium (CTAB) or upon contact on the surface of the coating (DODAB) [19]. In pharmaceutics, polymers are often be used as reservoirs of the active principle or drug so that the polymer can control the release of the active molecule over time in vivo [3,67,68].

DODAB BF reduced the viability of *E. coli* by 2 logs (Figure 5c), whereas PMMA/DODAB NPs reduced viability by 0.5 logs CTAB (Figure 5d). Possibly, the lack of mobility of the DODAB BF across the bacterial cell wall to reach the cell membrane resulted in the much lower activity of DODAB in comparison to the one of CTAB. In fact, no leakage of intracellular contents was detected for DODAB BF interacting with *E. coli* in comparison to the pronounced leakage determined for CTAB and the anionic sodium dodecylsulfate (SDS) [29].

The high affinity of DODAB for PMMA determined its incorporation in the NPs, which was 0.7 mM remaining 1.3 mM in the supernatant after dialysis and centrifugation (Table 5). For the PMMA/DODAB NPs, the activity was lower than the one of DODAB BF (Figure 5c,d). A possible explanation for this is that the DODAB incorporated in the NPs was not leaving them to kill the microbia; this meant that only DODAB BF outside the NPs was effective.

**Figure 6:**
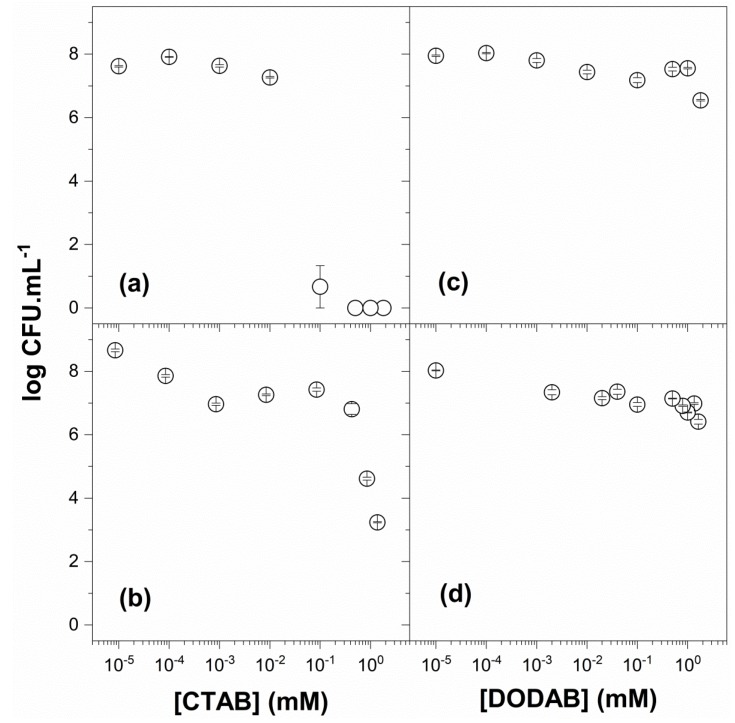
Viability of *Staphylococcus aureus* in the presence of CTAB (**a**), PMMA/CTAB (**b**), DODAB (**c**), and PMMA/DODAB dispersions (**d**). The NP dispersions of PMMA/CTAB and PMMA/DODAB were used after dialysis. The counting of colony-forming unities (CFU) was expressed on a logarithmic scale.

Figure 6 shows the compared activity of CTAB (Figure 6a) and PMMA/CTAB NPs (Figure 6b) against *S. aureus*. Similar to the effect of PMMA/CTAB NPs against *E. coli*, before dialysis there was 0.3 mM CTAB outside the PMMA/CTAB NPs with 1.7 mM incorporated in the NPs. The CTAB reservoir effect of the NPs reduced its effect against the bacteria, as compared to free CTAB. The compared effect for DODAB BF (Figure 6c) and PMMA/DODAB NPs (Figure 6d) revealed a similar and low activity. *Staphylococcus* sp. developed a sensor system for cationic antimicrobial peptides based on a sensor consisting of a short and negatively charged extracellular loop of amino acid residues able to interact with cationic antimicrobial peptides [69]. The transduction of this interaction signal would trigger the d-alanylation of teichoic acids and the lysylation of phosphatidylglycerol, resulting in a decreased negative charge of the bacteria. It is possible that DODAB BF (but not CTAB molecules) were possibly able to trigger this resistance mechanism in *S. aureus* due to the multipoint attachment of DODAB BF to the negatively charged loop. CTAB, on the other hand, would kill microorganisms as individual molecules below its critical micellar concentration (1 mM) [28,30], rapidly and extensively penetrating the cell wall to reach the cell membrane.

**Figure 7:**
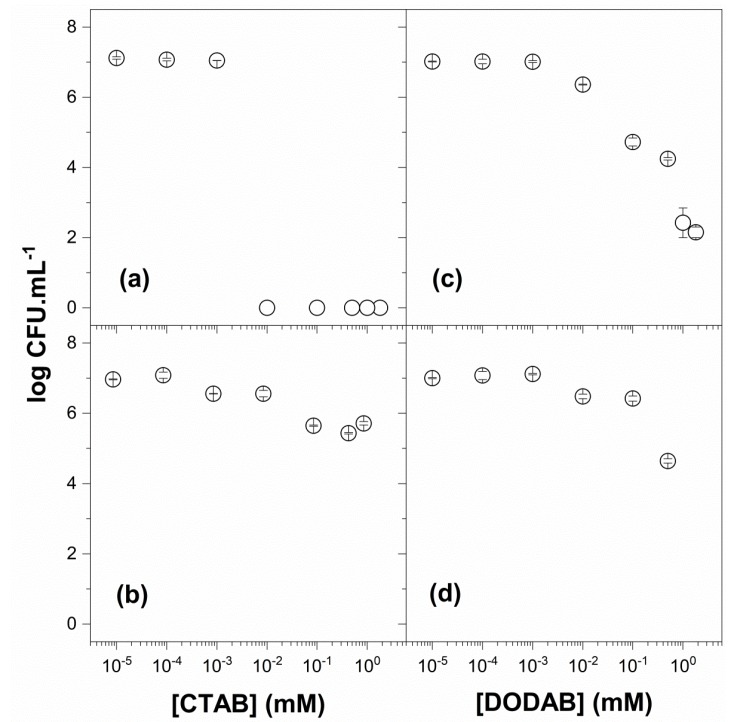
Viability of *Candida albicans* in the presence of CTAB (**a**), PMMA/CTAB (**b**), DODAB (**c**), and PMMA/DODAB dispersions (**d**). The NP dispersions of PMMA/CTAB and PMMA/DODAB were used after dialysis. The counting of colony-forming unities (CFU) was expressed in a logarithmic scale. The superior activity of QACs by themselves as compared to QACs in NPs is depicted from comparisons between (**a**,**b**) or (**c**,**d**). CTAB was active as a monomer at submicellar concentrations (**a**). DODAB was active as bilayer fragments (**c**).

Against *Candida albicans*, free CTAB showed remarkable activity (Figure 7a) in contrast with the reduced activity of PMMA/CTAB NPs (Figure 7b). The NP dispersion just after dialysis was used to interact with the fungus so that no CTAB molecules were available outside the NPs to interact with the cells. However, some CTAB leakage from the NPs was probably taking place to yield the 1.5-logs reduction observed in Figure 7b. On the other hand, DODAB BF was surprisingly active against *C. albicans*, resulting in a reduction of ca. 5 logs at 1.2 mM DODAB after a 1-h interaction (Figure 7c). Neither Campanhã and coworkers [40] nor Vieira and coworkers [31] could observe the good antifungal activity of DODAB BF since they employed percentiles of viable cells to express the viability curves as a function of DODAB concentration. Consistently, Fukushima and coworkers [70] also reported a superior activity of supramolecular assemblies made of poly(lactide) (interior block) and cationic polycarbonates (exterior block); upon testing the spherical and rod-like morphologies for antimicrobial properties, they found that only the rod-like assemblies were effective against *Candida albicans*. This showed that the shape of the antimicrobial supramolecular assembly was important to determine antifungal activity. In the present case, the disk-like shape of the DODAB BF assemblies resulted in good antifungal activity that was not described before (Figure 7c). This can possibly be ascribed to the improved penetration of rod-like or disk-like nanostructures through the outer layer of glicoproteins on the fungus cell wall as compared to vesicles, liposomes, or nanoparticles [42,52,71].

In Figure 7d, the reduced activity of PMMA/DODAB NPs in comparison to the one of DODAB BF (Figure 7c), again, can be understood from the incorporation of DODAB in the NPs reducing its availability to interact with the fungus; there was 1.3 mM DODAB BF outside the NPs, whereas, in the DODAB BF, dispersion was 2.0 mM DODAB (Figure 7c; Table 5).

**Figure 8:**
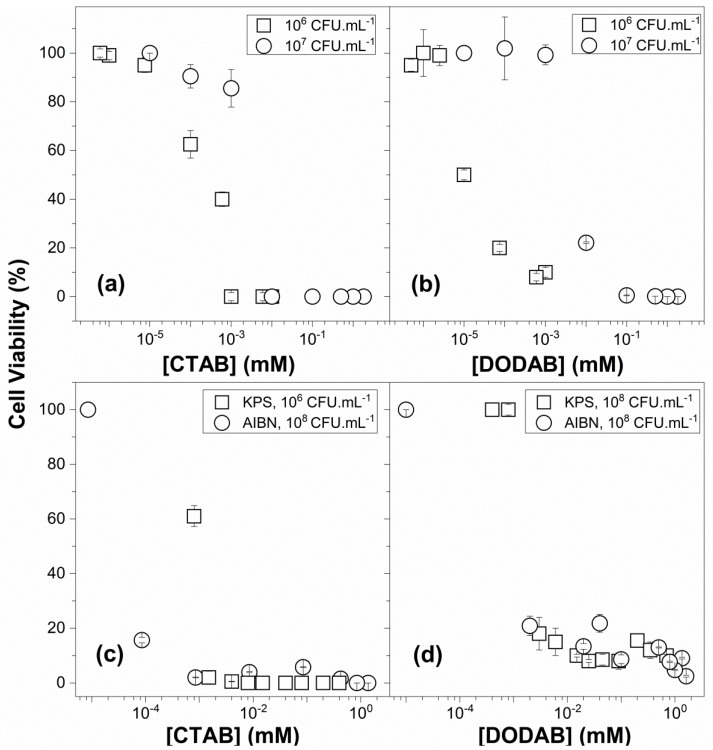
*Candida albicans* viability (%) (○) over a range of CTAB (**a**) or DODAB concentrations (**b**) in QAC dispersions in water as compared to data from [31] (□). *Staphylococcus aureus* viability (%) (○) over a range of CTAB (**c**) or DODAB concentrations (**d**) in the presence of PMMA/QAC NPs, as compared to data from [27] (□). QAC dispersions in water are CTAB micelles or DODAB BF. In (**c**,**d**), PMMA/QAC NPs were obtained by the emulsion polymerization of MMA in the presence of QACs. These NPs were obtained either using AIBN (○) or KPS (□) as the initiator. [MMA] and [QAC] used during particle synthesis were 0.56 M and 4.0 mM, respectively, for squares in (**c**), 0.4 M and 2.0 mM, respectively, for circles in (**c**,**d**), and 0.56 M and 10 mM, respectively, for squares in (**d**).

In Figure 8, the cell viability of *Candida albicans* as a percentile of viable cells (%) was obtained as a function of CTAB (Figure 8a) or DODAB concentration (Figure 8b) and compared with previous data from the literature [31]. There was a dependence of the viability curve on the initial concentration of viable cells: around 10^6^ CFU.mL^−1^, the sigmoidal curve occupied a range of lower QAC concentrations than the one around 10^7^ CFU.mL^−1^. This was consistent with the lower amounts of QAC required to kill lower concentrations of cells. In addition, one should notice that achieving a reduction of two logs in viable cells counting did not allow to discriminate whether the effect of the QACs reduced the counting by more than 2 logs. It is necessary to express CFU counting on a logarithmic scale in order to determine the complete effect of any antimicrobial, as done against *C. albicans* in Figure 7a,c. Consequently, the CTAB and DODAB BF effects were fully revealed, showing important reductions in CFU countings over 7 and 5 logs, respectively (Figure 7a,c).

Regarding the PMMA/CTAB NPs, the percentiles of viable countings plotted as circles in Figure 8c did not reveal the 5-logs reduction observed against *S. aureus* in Figure 6b. Only in the case of a small antimicrobial effect were the percentiles of viable cells sufficient to determine antimicrobial activity as was the case of the 1.0-logs reduction caused by PMMA/DODAB NPs in Figure 8d corresponding to Figure 6d expressed on a logarithmic scale.

## 4. Conclusions

Over a range of MMA or QAC concentrations, 0.4 M MMA and 2 mM QAC yielded optimal physical properties for the PMMA/DODAB and PMMA/CTAB NPs in dispersion, such as nanometric size (Dz < 100 nm), low polydispersity (<0.05), high and positive zeta-potential (>20 mV), high yield (>70%), high particle number density (>10^13^ particles.mL^−1^), and high colloidal stability (absence of aggregates and precipitates). Among two initiators (KPS or AIBN), AIBN was the best one to obtain optimal properties for the NPs synthesized at 2 mM QAC and 0.56 M MMA; the low sizes, high positive zeta-potentials, and low polydispersities occurred only using AIBN.

Inhibition halos by CTAB and PMMA/CTAB NPs against bacteria and yeast showed that PMMA/CTAB NPs behaved as reservoirs for the release of CTAB with time after dialysis; CTAB was able to move both through the dialysis membrane and the agar to inhibit microbial growth, highlighting hydrogels as good vehicles for CTAB. In contrast, DODAB preferred to remain incorporated in the PMMA polymeric matrix, did not move in the agar, and did not cross the dialysis bag membrane; for PMMA/DODAB NPs or DODAB BF, inhibition halos were not observed due to the lack of DODAB BF diffusion in the agar medium.

The incorporation of QACs in the PMMA/QAC NPs reduced antimicrobial activity in comparison to the QACs dispersions. CTAB was the most active microbicidal agent against the three microbes tested (*E. coli*, *S. aureus*, and *C. albicans*), reducing cell viability countings by 7 logs at submicellar concentrations. The controlled release of CTAB from PMMA/CTAB NPs, however, would be a promising strategy for CTAB delivery. CTAB mobility in hydrated medium favored its electrostatic interaction and penetration through the microbes’ cell wall and membrane, imparting a lytic effect on the cells. DODAB BF revealed an important fungicidal activity against *C. albicans* not described before; similar to rod-like, cationic supramolecular assemblies, the disk-like cationic DODAB BF possibly entered the outer glycoproteins layer of the fungus penetrating the cell and causing a 5-logs reduction in yeast viability.

The real potency of QACs antimicrobials should not be evaluated in percentiles of initial CFU counting; potent agents often display reduction in microbial cell viability larger than the 2 logs of the initial counting of viable cells.

The PMMA/QAC NPs here described still require further evaluation regarding their cytotoxicity against mammalian cells before fulfilling their potential in drug and vaccine delivery. Due to their nanometric size, positive zeta-potential, narrow size distribution, and high colloidal stability, they are expected to combine well with oppositely charged antigens (proteins, peptides or DNA) for subunit vaccines’ design, as many cationic adjuvants do. CTAB leakage and lytic property against eukaryotic cells, such yeast *C. albicans*, would not recommend the PMMA/CTAB NPs for vaccines. DODAB, on the other hand, remains embedded in the PMMA matrix, imparting a positive charge to the NPs without leaking to the outer medium. These considerations suggest that PMMA/DODAB NPs should be kept in perspective for further testing and prospective uses in drug and vaccine delivery.

## Figures and Tables

**Table 1:** Dry mean diameter (D) and mean hydrodynamic diameter (Dz) for PMMA/QAC NPs. D was from scanning electron microscopy (SEM) and Dz was determined by dynamic light-scattering (DLS). The nanoparticles (NPs) were obtained from 0.4 M methyl methacrylate (MMA), 2 mM dioctadecyl dimethyl ammonium bromide (DODAB) or cetyl trimethylammonium bromide (CTAB), and 0.36 mg·mL^−1^ AIBN in 10 mL of 1 mM NaCl. The mean D was obtained using ImageJ software from 100 particles.

NPs	D (nm)	Dz (nm)
PMMA/DODAB	56 ± 7	75 ± 1
PMMA/CTAB	85 ± 11	81 ± 1

**Table 2:** Physical properties of PMMA/QAC NPs obtained by emulsion polymerization of MMA monomer over a range of [MMA] at 2 mM DODAB or CTAB using AIBN as the initiator. Zeta-average diameter (Dz), zeta potential (ζ), and polydispersity (P) were obtained by dynamic light scattering. The conversion of monomer into polymer was expressed in percentile. The particles’ number density (N_p_), in mL^−1^, was calculated from nanoparticle size (Dz) and the mass of lyophilized dispersions. Solid contents, conversion, and N_p_ were determined as previously described in [27].

QAC	[MMA] /M	Dz /nm	ζ /mV	P	Solid Contents /mg·mL^−1^	Conversion /%	N_p_ /mL^−1^	Aggregates
DODAB	0.1	101 ± 1	+20 ± 2	0.355 ± 0.002	0.0051 ± 0.0001	51	8.35 × 10^12^	No
	0.2	52 ± 1	+20 ± 1	0.265 ± 0.003	0.0081 ± 0.0001	40	9.52 × 10^13^	No
	0.3	73 ± 1	+27 ± 1	0.038 ± 0.010	0.0183 ± 0.0009	61	7.76 × 10^13^	No
	0.4	75 ± 1	+49 ± 5	0.037 ± 0.005	0.0317 ± 0.0001	79	1.26 × 10^14^	No
	0.7	94 ± 1	+26 ± 2	0.033 ± 0.009	0.0479 ± 0.0008	68	1.04 × 10^14^	Yes
	0.9	103 ± 1	+38 ± 2	0.013 ± 0.004	0.0697 ± 0.0001	77	1.07 × 10^14^	Yes
	1.0	109 ± 1	+33 ± 1	0.020 ± 0.010	0.0781 ± 0.0005	78	1.02 × 10^14^	Yes
CTAB	0.1	916 ± 56	+18 ± 1	0.477 ± 0.042	0.0021 ± 0.0004	20	4.43 × 10^9^	No
	0.2	40 ± 1	+20 ± 1	0.291 ± 0.003	0.0105 ± 0.0004	53	2.82 × 10^14^	No
	0.3	70 ± 1	+17 ± 1	0.078 ± 0.009	0.0197 ± 0.0019	65	9.52 × 10^13^	No
	0.4	81 ± 1	+23 ± 1	0.041 ± 0.008	0.0320 ± 0.0019	80	1.01 × 10^14^	No
	0.7	100 ± 1	+20 ± 1	0.032 ± 0.008	0.0363 ± 0.0014	52	6.10 × 10^13^	Yes
	0.9	121 ± 1	+26 ± 1	0.012± 0.004	0.0692 ± 0.0013	77	6.52 × 10^13^	Yes
	1.0	121 ± 1	+17 ± 1	0.046 ± 0.010	0.0702 ± 0.0005	70	6.52 × 10^13^	Yes

**Table 3:** Physical properties of PMMA/QAC NPs obtained by the emulsion polymerization of MMA monomer at 0.4 M MMA over a range of [QAC] using AIBN as the initiator. Zeta-average diameter (Dz), zeta potential (ζ), and polydispersity (P) were obtained by dynamic light scattering. The conversion of monomer into polymer was expressed in percentile. The particles number density (N_p_), in mL^−1^, was calculated from nanoparticle size (Dz) and the mass of lyophilized dispersions.

QAC	[QAC] /mM	Dz /nm	ζ /mV	P	Solid Contents /mg·mL^−1^	Conversion /%	N_p_ /mL^−1^
DODAB	0.3	99 ± 1	+50 ± 2	0.035 ± 0.009	0.0303 ± 0.0003	76	5.24 × 10^13^
	0.5	93 ± 1	+44 ± 2	0.045 ± 0.014	0.0300 ± 0.0004	75	6.21 × 10^13^
	1.0	85 ± 1	+33 ± 2	0.028 ± 0.008	0.0303 ± 0.0006	76	8.08 × 10^13^
	2.0	75 ± 1	+49 ± 5	0.037 ± 0.005	0.0317 ± 0.0001	79	1.26 × 10^14^
	4.0	69 ± 1	+31 ± 1	0.072 ± 0.008	0.0349 ± 0.0006	87	1.79 × 10^14^
	5.0	62 ± 1	+29 ± 2	0.068 ± 0.010	0.0333 ± 0.0006	83	2.31 × 10^14^
	8.0	58 ± 1	+32 ± 2	0.098 ± 0.010	0.0365 ± 0.0008	91	3.04 × 10^14^
	10.0	59 ± 1	+35 ± 3	0.123 ± 0.007	0.0364 ± 0.0021	91	2.92 × 10^14^
CTAB	0.3	126 ± 1	+15 ± 1	0.055 ± 0.013	0.0261 ± 0.0001	65	2.16 × 10^13^
	0.5	115 ± 1	+15 ± 1	0.069 ± 0.016	0.0274 ± 0.0001	68	3.02 × 10^13^
	1.0	103 ± 1	+27 ± 2	0.069 ± 0.013	0.0319 ± 0.0005	80	4.86 × 10^13^
	2.0	81 ± 1	+23 ± 1	0.041 ± 0.008	0.0320 ± 0.0019	80	1.01 × 10^14^
	4.0	67 ± 1	+24 ± 1	0.070 ± 0.011	0.0282 ± 0.0003	70	1.55 × 10^14^
	5.0	67 ± 1	+31 ± 2	0.052 ± 0.012	0.0234 ± 0.0006	58	1.32 × 10^14^
	8.0	60 ± 1	+34 ± 1	0.095 ± 0.011	0.0320 ± 0.0002	80	2.50 × 10^14^
	10.0	57 ± 1	+41 ± 2	0.092 ± 0.008	0.0308 ± 0.0001	77	2.72 × 10^14^

**Table 4:** Comparison between nanoparticles of PMMA/QAC synthesized using either potassium persulfate (KPS) or azobisisobutyronitrile (AIBN) as initiators, at 0.56 M MMA and 2 mM QAC.

NPs	Initiator	Dz/nm	ζ/mV	P
PMMA/DODAB	KPS ^1^	1260 ± 43	−10 ± 1	0.370
	AIBN	89 ± 1	+45 ± 2	0.027 ± 0.010
PMMA/CTAB	KPS ^1^	395 ± 5	−38 ± 1	0.262
	AIBN	96 ± 1	+23 ± 1	0.033 ± 0.012

^1^ Results taken from the reference [27].

**Table 5:** Determination of QAC concentration [QAC] in PMMA/QAC NPs from halide microtitration in the supernatants of PMMA/DODAB and PMMA/CTAB NP water dispersions before and after dialysis. The PMMA/QAC dispersions were prepared in 2.0 mM QAC. The controls for the dialysis were QAC dispersions in water and 1 mM NaCl solution.

Dispersion or Solution	[QAC]/mM	
	Before Dialysis	After Dialysis
CTAB dispersion in water	2.5 ± 0.1	0.1 ± 0.1
DODAB bilayer fragments in water	2.3 ± 0.1	2.0 ± 0.1
NaCl water solution	1.2 ± 0.1	0.2 ± 0.1
Supernatant of PMMA/DODAB dispersion	2.0 ± 0.1	1.3 ± 0.1 ^1^
Supernatant of PMMA/CTAB dispersion	2.0 ± 0.1	0.5 ± 0.1 ^1^

^1^ Microtitration done for supernatants of dialyzed dispersions 3 days after dialysis.
